# Is the tumor cell side of the immunological synapse a polarized secretory domain?

**DOI:** 10.3389/fimmu.2024.1452810

**Published:** 2024-09-24

**Authors:** Andrea Michela Biolato, Liza Filali, Diogo Pereira Fernandes, Flora Moreau, Takouhie Mgrditchian, Céline Hoffmann, Clément Thomas

**Affiliations:** ^1^ Cytoskeleton and Cancer Progression, Department of Cancer Research (DOCR), Luxembourg Institute of Health (LIH), Luxembourg City, Luxembourg; ^2^ Faculty of Science, Technology and Medicine (FSTM), University of Luxembourg, Esch-sur-Alzette, Luxembourg; ^3^ National Cytometry Platform (NCP), Luxembourg Institute of Health (LIH), Esch-sur-Alzette, Luxembourg

**Keywords:** immunological synapse, natural killer (Nk) cell, actin cytoskeleton, multivesicular bodies (MVB), cancer, targeted secretion

## Abstract

The formation of a lytic immunological synapse (IS) is crucial for cytotoxic lymphocytes to accurately target and effectively eliminate malignant cells. While significant attention has been focused on the lymphocyte side of the IS, particularly its role as a secretory domain for lytic granules, the cancer cell side of the IS has remained relatively underexplored. Recent findings have revealed that cancer cells can rapidly polarize their actin cytoskeleton toward the IS upon interaction with natural killer (NK) cells, thereby evading NK cell-mediated cytotoxicity. In this Brief Research Report, we present preliminary findings suggesting that actin cytoskeleton remodeling at the cancer cell side of the IS is associated with the targeted secretion of small extracellular vesicles towards the interacting NK cell. We observed that multivesicular bodies (MVBs) preferentially accumulate in the synaptic region in cancer cells exhibiting synaptic accumulation of F-actin, compared to those lacking actin cytoskeleton remodeling. Extracellular immunofluorescence staining revealed increased surface exposure of CD63 at the cancer cell side of the IS, suggestive of the fusion of MVBs with the plasma membrane. This hypothesis was supported by a pH-sensitive probe demonstrating dynamic trafficking of CD63 to the extracellular region of the IS. Collectively, our data support the notion that cancer cells can engage in targeted secretion of extracellular vesicles in response to NK cell attack, underscoring the need for further research into the potential role of this process in facilitating cancer cell immune evasion.

## Introduction

The eradication of cancer cells by cytotoxic lymphocytes (CLs), such as cytotoxic T cells (CTLs) and natural killer (NK) cells, relies on the formation of a specialized cellular interface known as the lytic immunological synapse (IS). The IS serves as a platform for integrating signals present on the surface of potential target cells. Upon activation of CL cytolytic effector function, the IS facilitates the recruitment of lytic granules, also known as secretory lysosomes. The polarized secretion of lytic granules and the subsequent release of cytotoxic effector molecules, such as perforin and granzymes, into the synaptic cleft facilitate the precise and effective eradication of target cells, while minimizing collateral damage to surrounding tissues. In addition to effector molecules, T cells also exhibit polarized secretion of extracellular vesicles (EVs), including but not limited to exosomes derived from multivesicular bodies (MVBs) (1–6). A novel platform based on bead-supported lipid bilayers was recently developed to isolate trans-synaptic EVs secreted by T cells, including CD8+ CTLs, and directly compare their properties and composition to EVs constitutively secreted by the same cells. Trans-synaptic EVs were found to be larger, have a higher secretion rate, and possess a distinct composition compared to constitutively secreted EVs, underscoring their specialized role as trans-synaptic effectors (7).

Although cancer cells are known to secrete large quantities of exosomes with significant immunomodulatory effects (8, 9), it remains uncertain whether these cells can specifically redirect exosome secretion towards physically interacting cytotoxic lymphocytes. Nevertheless, if cancer cell-derived exosomes were to be secreted directionally into the synaptic cleft and reach elevated concentrations, this could potentially amplify their immunomodulatory impact on cytotoxic lymphocytes, thereby promoting immune evasion.

In lymphocytes, the establishment of an IS along with its associated secretory domain involves substantial reorganization of the actin cytoskeleton (10). Following an initial burst of actin polymerization at the contact region with the target cell, the synaptic actin network reorganizes into a peripheral F-actin-rich region and a central F-actin-hypodense region (11, 12). Secretory vesicles then polarize along the centrosome towards the F-actin-poor central region before releasing their cytotoxic effectors or stimulatory factors (e.g. cytokines), depending on the type of IS, as well as exosomes (13, 14). Moreover, in NK cells, nanoscale dynamism of the fine cortical branch F-actin mesh at the IS center has been shown to be indispensable for generating permissive F-actin clearances that allow granules to access the plasma membrane and facilitate secretion (15). This further underscores the pivotal role of the actin cytoskeleton in regulating polarized secretion.

We previously reported that a subset of cells derived from diverse breast cancer cell lines respond to NK cell synapsing by rapidly polarizing their filamentous actin cytoskeleton towards the IS, a process we termed an actin response (AR) (16). Although the underlying mechanism remains unclear, synaptic F-actin remodeling was causally associated with breast cancer cell resistance to NK cell-mediated cytotoxicity, as demonstrated by live cell imaging analyses. Consistent with these findings, complementary analyses showed reduced levels of granzyme B and apoptosis in NK cell-conjugated target cells exhibiting an AR. This actin-dependent synaptic evasion mechanism was also observed in chronic lymphocytic leukemia (CLL) cell lines and primary CLL cells from patients, suggesting its conservation across different malignancies (17). Notably, pharmacological inhibition of actin cytoskeleton remodeling, combined with an anti-HLA-G blocking antibody to facilitate conjugation, reduced primary CLL cell resistance to NK cell-mediated killing. This highlights the potential of targeting the AR to enhance anti-tumor immunity.

Given the crucial role of actin in establishing and maintaining the secretory domain at the lymphocyte cell side of the IS (11), we wondered whether actin cytoskeleton remodeling in cancer cells could be associated with the synaptic polarization of a vesicular compartment and the directed secretion of cancer cell-derived EVs towards the interacting NK cells. The preliminary findings outlined in this Brief Research Report extend our understanding of the lytic IS and suggest a broader functionality beyond its conventional role as a one-way secretory channel for CLs to release cytotoxic molecules and EVs towards target cells. Our investigations show that actin cytoskeleton remodeling at the cancer cell side of the IS is associated with significant enrichment in MVBs in the same region and fusion of some of these MVBs with the plasma membrane.

## Materials and methods

### Cell lines and culture

The human breast adenocarcinoma cell line MDA-MB-231 and the human melanoma cell line A-375 were purchased from ATCC (CRM-HTB-26™, CRL-1619™). These cell lines were cultured in DMEM medium, supplemented with 10% fetal bovine serum (FBS) and 1% Penicillin/Streptomycin (Thermo Fisher Scientific). The human malignant non-Hodgkin’s lymphoma NK-92 MI effector cell line (CRL-2408™, ATCC) was cultured in RPMI 1640 medium, supplemented with 10% FBS, 10% Horse Serum and 1% Penicillin/Streptomycin (Thermo Fisher Scientific). All cell lines were maintained in a humidified atmosphere at 37°C/5% CO_2_ and regularly screened for Mycoplasma contamination. Authentication of each cell line was performed, confirming their identity and absence of cross-contamination through STR profiling analysis (Mycrosynth).

### Cell transduction

MDA-MB-231 and A-375 cell lines were transduced to express the F-actin reported Emerald-LifeAct (LifeAct) (18) as previously described (16, 17). Briefly, the mEmerald-LifeAct-7 fragment (Addgene, #54148) was subcloned into the viral pCDH-EF1a-MCS-IRES-puro plasmid (System Biosciences) and used to produce infectious particles in HEK293 cells. Transduced cells were selected with puromycin (0.5 μg/ml, Sigma-Aldrich). MDA-MB-231 LifeAct cells were transduced to express the pHluorin-CD63-mScarlet protein. PLenti-pHluorin_M153R-CD63-mScarlet was a gift from Alissa Weaver (Addgene, # 172118). The plasmid was used to produce infectious particles in HEK293 cells. Transduced cells were selected with blasticidin (2 µg/mL, Santa Cruz). Following selection, the cells were sorted to obtain a population with similar expression levels.

### Fluorescence staining and confocal microscopy

NK-92 MI cells were labeled if necessary with CellTracker™ Deep Red (1μM, Thermo Fisher Scientific) in serum-free medium prior to co-culturing with target cells at an Effector: Target (E:T) ratio of 1:1. Cells were conjugated for 40 minutes at 37°C/5% CO_2_ and subsequently transferred onto a Poly-L-Lysine-coated μ-slide (ibidi) for 10 minutes before fixation with 2% paraformaldehyde (PFA, Agar scientific) for 15 minutes at room temperature (RT). Cells were permeabilized with 0.1% Triton X-100 (Sigma Aldrich) and labelled with anti-CD63 mouse antibody (1 μg/mL, Novus, clone MEM-259) and anti-CD9 rabbit antibody (1.5 μg/mL, Novus, clone SA35-08) for 1 hour at RT. Cells were then washed with PBS and incubated with secondary goat antibody Alexa Fluor 555 or Alexa Fluor 633 (Invitrogen) and DAPI (0.2 mg/mL, Sigma-Aldrich). After 2 washing steps, PBS was replaced by mounting medium (ibidi) before cell imaging. For extracellular labelling, the anti-CD63 antibody was added at a concentration of 2 µg/mL during the 40-minutes conjugation step. Following fixation, extracellular CD63 was detected using a secondary antibody (goat anti-mouse Alexa Fluor 555) without permeabilization. MDA-MB-231 pHluorin-CD63-mScarlet cells were pre-incubated with 1 μM SiR-Actin (Spirochrome, #SC001) in complete medium for 1 hour at 37°C/5% CO_2_ and then conjugated with unlabeled NK-92 MI cells at an E:T ratio of 1:1. Cells were conjugated for 20 minutes in a μ-slide chamber and maintained under the microscope at 37°C/5% CO2.

Images were acquired on a Zeiss LSM880 fastAiry confocal microscope with excitation laser including wavelengths at 405 nm, 488 nm, 543 nm, and 633 nm configured in a multitrack setup. For CD63 quantification, we generated a stack of the entire target cell at 0.5 μm intervals between slices, resulting in 20 to 30 slices per cell, depending on the cell size. This approach ensured that all CD63-positive vesicles were captured within the total cell volume. For high-resolution images, the Airy mode was employed. A stack with a 0.2 μm interval was captured, followed by subsequent deconvolution using the Airyscan joint deconvolution software (jDCV) within the Zen Blue v3.5 software. For live imaging of pHluorin-CD63-mScarlet localization, the pinhole was set to capture a 2 μm-depth slice. Stacks consisting of 5 slices with a 2 μm interval were acquired. Plot profiles were generated using ImageJ v1.53t software. A 60-pixel line was drawn across the conjugate, and the fluorescence intensity of each fluorophore was measured along this line. The ratio of pHluorin or actin at the synapse was calculated as the maximum intensity at the IS divided by the maximum intensity at the opposite side of the target cell.

The quantification of CD63 synaptic enrichment was performed using ImageJ v1.53t software. For each stack, a Z-projection was generated using the ‘sum slices’ method to compile information from all the Z-slices. A rectangular selection was manually delineated on the target cell within the conjugate. This selection was automatically partitioned into three regions of interest (ROIs): the synaptic region, the intermediate region, and the distal region, each corresponding to one-third of the target cell (**Supplementary Figure 1**). The mean pixel intensity (MPI) of LifeAct and CD63 was calculated in the synaptic and distal ROIs. The enrichment at the immunological synapse (IS) was determined by calculating the ratio of the MPI in the synaptic region to the MPI in the distal region for LifeAct and CD63.

### Fluorescence staining and imaging flow cytometry

NK-92 MI cells were labeled with anti-human CD56-PE/Cy7 (BioLegend, clone HCD56) prior to co-culturing with LifeAct target cells at an E:T ratio of 3:1 in the presence of Hoechst 33342 (0.5 mg/mL final; Miltenyi Biotec). Cells were allowed to conjugate for 40 minutes at 37°C, then fixed with 2% paraformaldehyde (PFA, Agar Scientific) for 15 minutes at room temperature (RT) and permeabilized with 0.1% Triton X-100 for 5 minutes. Cells were then stained with anti-human CD63-Alexa Fluor-594 (BioLegend, clone H5C6), anti-human CD9-PE (BioLegend, clone HI9a) and anti-human CD81-APC (BioLegend, clone 5A6) for 1 hour at RT. Following staining, cells were washed twice with PBS and resuspended in 35 µL.

Samples were acquired on an ImageStream^®^X Mark II (Cytek Biosciences) with four built-in lasers (405 nm, 488 nm, 561 nm, 642 nm). Using INSPIRE^®^ software (Cytek Biosciences), a total of 5x10^4^ E/T conjugates were captured per tube at a magnification of 60X using low-speed and high-sensitivity settings. Gating strategies, masks, and features were developed and applied for the analysis of conjugates as previously detailed (19). Briefly, the synaptic mask, overlaying the synaptic region, was created using Boolean logic involving 2 channels, LifeAct (ch02) and CD56-PE/Cy7 (ch06). A mask covering the remaining portion of the cell; the non-synaptic mask was also generated. For enrichment, the MPI of the protein of interest was calculated in both masks. Enrichment was calculated as the ratio of MPI within the synaptic mask versus the non-synaptic mask. Target cells were classified as AR- or AR+ according to synaptic enrichment values for F-actin (Emerald-LifeAct), with AR- defined as having values <1 and AR+ defined as having values >1. This classification was subsequently validated through visual inspection to ensure accuracy and exclude false positives and negatives. For CD63, CD9 or CD81, the total intensity was calculated within their respective masks that cover the entire target cell.

### Statistical analysis

Statistical analysis was performed using GraphPad Prism software (version 10.8.1) to determine the statistical significance of the observed distinctions among groups. A Shapiro-Wilk test was used to assess normality of data. Since data often deviate from normality, we opted for a non-parametric Mann-Whitney, Wilcoxon or Kruskal-Wallis test. Specific statistical tests employed are detailed in the respective figure legends.

## Results

**Figure 1:**
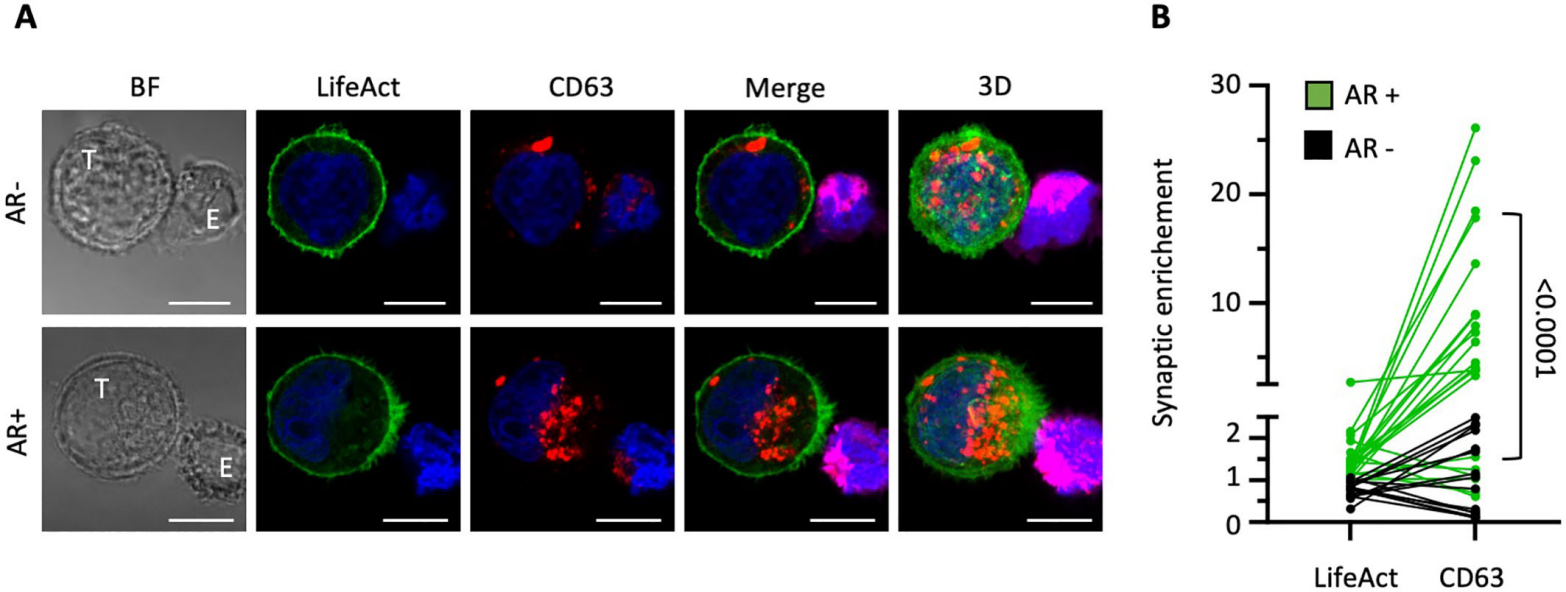
Polarization of the cancer cell actin cytoskeleton to the immunological synapse is associated with a local increase in multivesicular bodies. MDA-MB-231 target cells (T) expressing the actin reporter Emerald-LifeAct (green) were co-cultured with NK-92 MI effector cells (E) for 40 minutes and stained for CD63 (red). **(A)** Representative confocal microscopy images of conjugates between MDA-MB-231 cells (green; T) and DeepRed-stained NK-92 MI cells (purple; E). The top row shows a cell-to-cell conjugate without synaptic actin enrichment at the cancer cell side of the immunological synapse (actin response; AR-). The bottom row displays a conjugate with an actin response (AR+). The three-dimensional images on the right (3D) are projections of 22 and 34 confocal sections of 0.5 µm. Scale bars: 10 µm. **(B)** Synaptic enrichment of F-actin (LifeAct) and CD63 in AR+ and AR- target cells. A total of 36 cell-to-cell conjugates were analyzed from two independent experiments. Statistical significance was assessed using the Mann-Whitney test.

In our prior work, we established that cancer cells can respond to direct contact with NK cells by rapidly polarizing F-actin toward the synaptic junction, allowing them to evade destruction, a process we termed “actin response” (AR) (16, 17). However, the potential link between actin cytoskeleton remodeling and membrane/vesicle trafficking at the tumor cell side of the IS has not been carefully evaluated so far. To address this gap, we challenged MDA-MB-231 breast carcinoma cells with NK92-MI cells, and assessed the relative distribution of the CD63-labelled vesicular compartment in NK cell-conjugated MDA-MB-231 cells exhibiting or not exhibiting synaptic accumulation of F-actin. To specifically visualize the actin cytoskeleton at the target cell (postsynaptic) side of the IS and avoid interference from the NK cell cytoskeleton, we stably introduced the fluorescent F-actin reporter Emerald-LifeAct into MDA-MB-231 cells, as detailed previously (16). As illustrated in **Figure 1A**, the CD63 signal exhibited a significant trend towards enrichment near the IS in target cells with an AR, contrasting with a more random distribution across cells lacking synaptic F-actin accumulation. To quantify F-actin and CD63 synaptic enrichment, we compared the relative intensity of each signal in a synaptic region covering approximately one third of the cell with a similarly sized region on the opposite side (**Supplementary Figure 1A**). Our analysis revealed that cells with an AR showed more frequent and pronounced synaptic enrichment of CD63 within the IS, with an average fold difference of 7.8 ± 1.7 (n=21; **Figure 1B**). In contrast, cells lacking an AR demonstrated less frequent and only modest CD63 synaptic enrichment, with an average fold difference of 1.1 ± 0.2 (n= 15). Thus, accumulation of filamentous actin at the cancer cell side of the IS is associated with a significant enrichment of the CD63-positive vesicular compartment in the synaptic region (p<0.0001).

**Figure 2:**
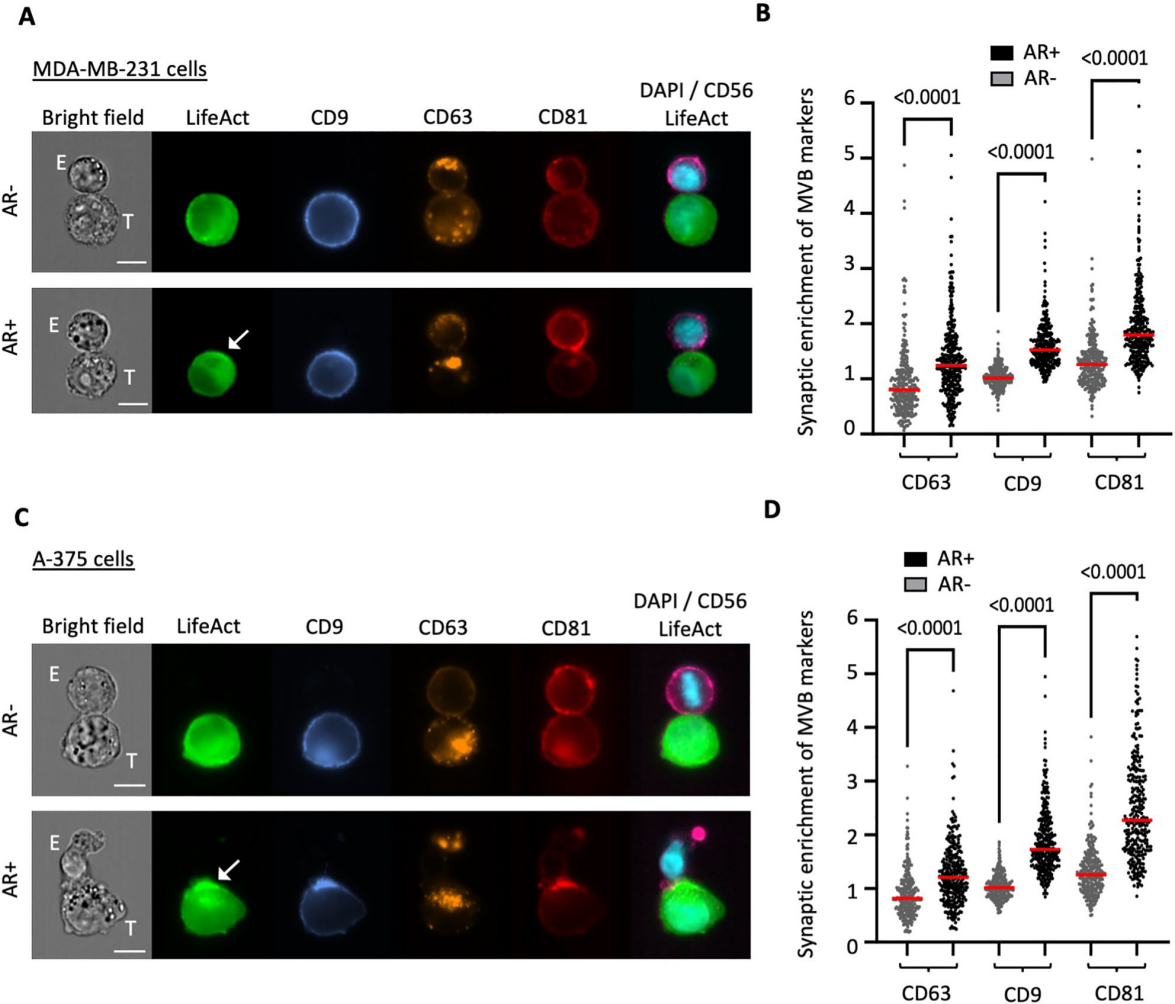
Throughput analysis of synaptic enrichment of MVB markers in relation to the actin cytoskeleton using imaging flow cytometry (IFC). Emerald-LifeAct-expressing MDA-MB-231 cells or A-375 target cells (T) were co-cultured with CD56-stained NK-92 MI effector cells (E) for 40 minutes and immunolabeled for the tetraspanin proteins CD9 (blue), CD63 (orange) and CD81 (red). **(A, C)** Representative IFC panels of conjugates between MDA-MB-231 **(A)** or A-375 **(C)** target cells and NK-92 MI effector cells. The top rows depict conjugates without actin response (AR-), while the bottom rows show conjugates with an actin response (AR+). Scale bar: 10 µm. **(B, D)** Quantitative analysis of synaptic enrichment of CD63, CD9 and CD81 in actin response-negative (AR-) and actin response-positive (AR+) MDA-MB-231 **(B)** or A-375 **(D)** target cells. A total of 300 conjugates were analyzed in three independent experiments. The red line indicates the mean value, and the statistical significance was assessed using a Kruskal-Wallis test.

Imaging flow cytometry was utilized to validate the aforementioned findings across a larger cohort of conjugates and to expand our analysis to encompass additional classical markers of cancer cell-derived extracellular vesicles (EVs), including CD9 and CD81. Contrary to CD63, which predominantly localized intracellularly, both CD9 and CD81 displayed notable localization at the plasma membrane alongside their intracellular presence (**Figure 2A**). However, their distribution was not uniform at the cell periphery, as evidenced by regions of heightened intensity, potentially indicating their enrichment in EVs. The synaptic enrichment of F-actin and the three tetraspanins was evaluated by employing synaptic and non-synaptic masks and determining the ratio of mean pixel intensity (MPI) within each mask, as depicted in **Supplementary Figure 1B** and further detailed recently (19). Consistent with our previous report (16), MDA-MB-231 cells demonstrated a high capacity to remodel their actin cytoskeleton and assemble an AR upon NK cell attack, with approximately 46% of NK cell-conjugated cells showing synaptic polarization of F-actin (**Supplementary Figure 2A**). Our results revealed a significantly higher synaptic enrichment of the three tetraspanins in target cells exhibiting an AR compared to those without an AR (**Figure 2B**; p values < 0.0001). As shown in **Supplementary Figure 3A**, no statistically significant differences were observed in the overall expression levels of any of the three tetraspanins between subpopulations of target cells with and without an AR. This suggests that the synaptic enrichment of tetraspanins is likely attributed to the redistribution of existing protein reservoirs.

We extended our investigation to a distinct cancer cell model by introducing Emerald-Lifeact into A-375 melanoma cells, which we used as a source of target cells. Melanoma is a particularly relevant model for synaptic secretion, as demonstrated by pioneering studies (20, 21). Moreover, melanoma is widely recognized as a tumor model that secretes elevated levels of EVs, including exosomes, with key roles in cancer progression. Compared to the breast carcinoma model, A-375 melanoma cells demonstrated a slightly higher capacity to respond to NK cell interactions, with approximately 51% of the NK cell-conjugated cells showing an AR (**Supplementary Figure 2B**). Consistent with our previous findings in breast cancer cells, quantitative analysis revealed a significantly increased synaptic abundance of the three tetraspanins in melanoma cells exhibiting an AR compared to those lacking cytoskeletal rearrangement (**Figures 2C, D**). Similar to the breast cancer cells, there were no differences in the overall expression levels of tetraspanins (**Supplementary Figure 3B**).

**Figure 3:**
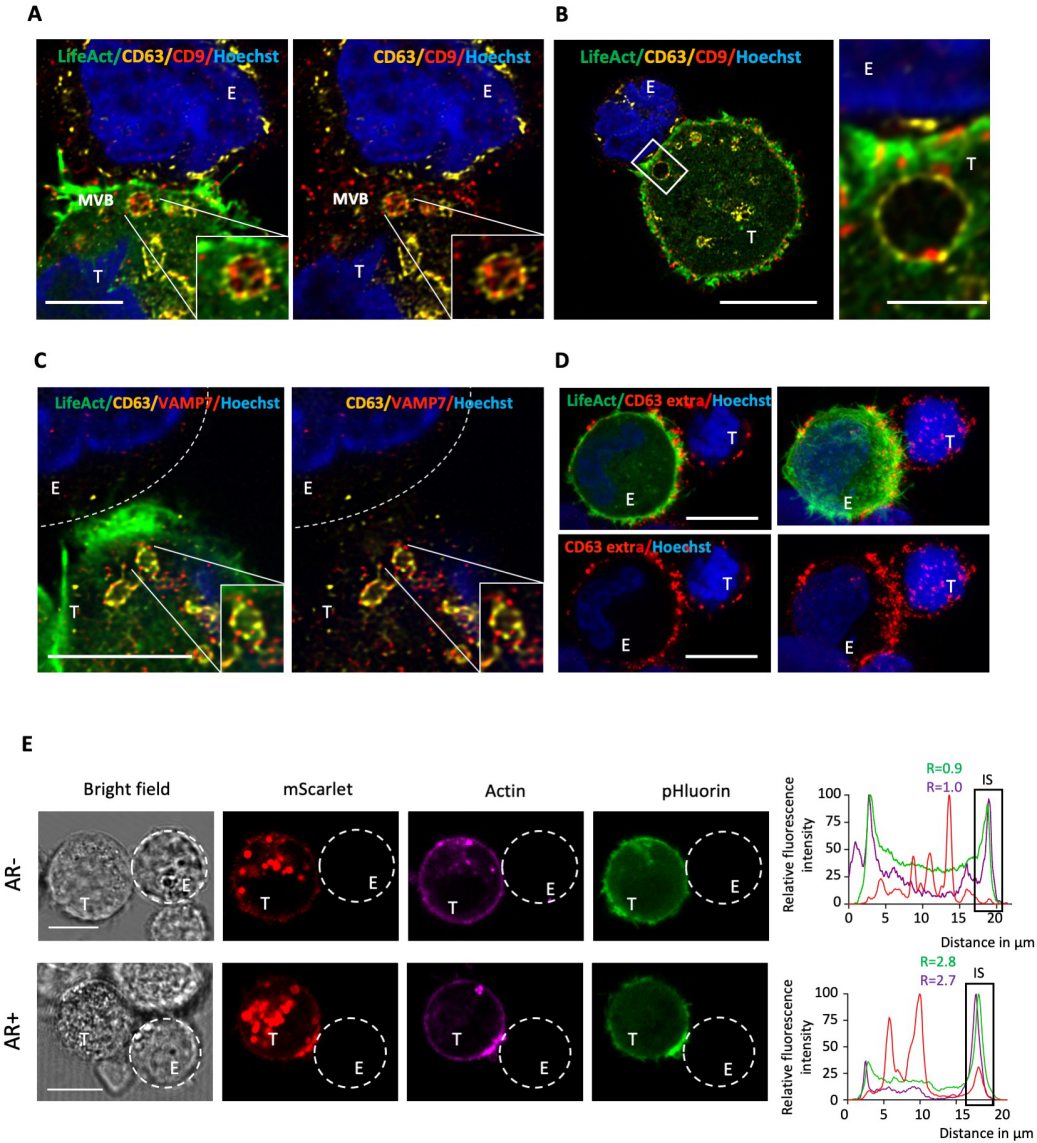
Super-resolution imaging of MVBs and CD63 exposure at the cancer cell side of the immunological synapse. **(A, B)** Emerald-LifeAct MDA-MB-231 target cells (T) were co-cultured with NK-92 MI effector cells (E) for 40 minutes and stained for the tetraspanin proteins CD63 and CD9. Super-resolution images display the co-localization of CD63 (yellow) and CD9 (red) within individual MVBs in the synaptic region of MDA-MB-231 cells with an actin response. Nuclei are stained in blue. Scale bars: 5 µm **(A)**, 10 µm (**B**, left panel) and 2 µm (**B**, right panel). **(C)** Super-resolution images showing co-localization of CD63 (yellow) and VAMP7 (red) within individual MVBs in the synaptic region of MDA-MB-231 cells with an actin response. Scale bar: 5 µm. **(D)** Emerald-LifeAct MDA-MB-231 cells (T) were co-culture with NK-92 MI effector cells (E) for 40 minutes in the presence of an anti-CD63 antibody. After fixation, a secondary antibody was used to visualize extracellular CD63 (red). Scale bar: 10 µm. **(E)** pHluorin-CD63-mScarlet MDA-MB-231 target cells (T) were co-culture with NK-92 MI effector cells (E). MDA-MB-231 cells were pre-labeled with the F-actin probe SiR-actin (purple). Red (mScarlet) and green (pHluorin) signals indicate the intracellular and extracellular fractions of CD63, respectively. The upper and lower panels show examples of cell-to-cell conjugates without and with an actin response (AR- and AR+), respectively. Scale bar: 10 µm. A 60-pixel-wide line was drawn across the longitudinal axis of cell-to-cell conjugates, and the fluorescence profile across the line was plotted. The relative fluorescence intensity (R) of actin (purple) and extracellular CD63 (green) in the synaptic region versus the opposite site of the target cell was calculated.

The synaptic enrichment of the three exosomal markers CD63, CD81, and CD9 prompted us to investigate the presence of multivesicular bodies (MVBs) within the synaptic region of target cells. LifeAct-expressing MDA-MB-231 cells were co-cultured with NK cells, concurrently labeled for CD63 and CD9, and subjected to imaging using the super-resolution (Airyscan) mode of our confocal microscope. Our data confirmed the presence of MVBs positive for both markers in close proximity to the IS in MDA-MB-231 cells undergoing synaptic actin cytoskeleton remodeling (**Figures 3A, B**), suggesting that directed secretion may occur from tumor cells towards NK cells. Moreover, synaptic MVBs were found to express VAMP7 (**Figure 3C**), a v-SNARE protein with a pivotal role in facilitating MVB fusion with the plasma membrane and subsequent exosome secretion in cancer cells (22, 23).

To obtain first insights into an increased extracellular exposure of MVBs at the cancer cell side of the IS, we performed extracellular staining of CD63 by introducing the antibody during co-culture of cancer cells and NK cells. This revealed a marked accumulation of extracellular CD63 within the synaptic area, evocative of a local elevated secretory activity (**Figure 3D**). However, the significant signal at the NK cell surface precluded definite conclusion about the origin of the CD63 extracellular signal. To tackle this issue and further examine the potential fusion of MVBs with the plasma membrane of target cells within the synaptic area, we employed the dual-color fluorescent reporter system pHluorin-CD63-mScarlet (24). This reporter is composed of two fluorophores: the pH-insensitive mScarlet, which emits red fluorescence consistently even under the acidic conditions of the MVB lumen, and the pH-sensitive pHluorin, which exhibits reduced fluorescence intensity in acidic conditions and emits green fluorescence upon exposure to the more neutral extracellular pH. The pH sensitivity of pHluorin-CD63-mScarlet in MDA-MB-231 cells was validated by increasing the intracellular pH using ammonium chloride (NH4Cl; **Supplementary Figure 4**). Prior their co-incubation with NK cells, target cells were labeled with the cell-permeable F-actin probe SiR-actin. NK cell-conjugated MDA-MB-231 cells lacking an AR displayed some green signal in various regions of their periphery, indicative of basal, non-polarized, CD63 exposure at the cell membrane (**Figure 3E**, upper raw). Conversely, NK cell-conjugated MDA-MB-231 cells exhibiting an AR demonstrated a pronounced polarization of the green signal toward the interacting NK cell, suggesting a redirection of secretion toward the IS (**Figure 3E**, lower raw).

In summary, our findings reveal that synaptic remodeling of the actin cytoskeleton within cancer cells engaged with NK cells is associated with an increased recruitment and fusion of MVBs at the IS. This suggests that cancer cell-derived exosomes may be released into the IS, raising important questions about the properties of these exosomes and their role in conferring resistance to NK cell-mediated cytotoxicity. Although further investigation is warranted, these insights highlight a potentially overlooked mechanism by which cancer cells might evade immune destruction.

## Discussion

Our observations reveal that the lytic IS formed between CLs and cancer cells is not merely a unidirectional secretion site for CL-derived lytic granules, EVs and non-vesicular killing particles, such as supramolecular attack particles (SMAPs) (6, 25). In certain instances, cancer cells quickly respond to IS formation by polarizing their actin cytoskeleton toward the IS (16, 17, 26), a process we found to be associated with local enrichment of EV-containing MVBs. The fusion of these MVBs with the cancer cell membrane suggests that exosomes are released into the synaptic cleft. Supporting the idea that the cancer cell side of the IS functions as a secretory domain, we have occasionally observed cancer cells with F-actin configurations resembling the characteristic radial organization seen at the CL side of the IS, consisting of a peripheral F-actin dense region and a central region with reduced F-actin density. Although this radial topology indirectly suggests the presence of a secretory domain, further investigations are necessary to precisely delineate the spatiotemporal organization of actin filaments in relation to MVBs at the cancer cell side of the IS.

Synaptic cancer cell-derived EVs may play a crucial role in influencing the outcome of the lytic IS. For example, previous studies have demonstrated that exosomal PD-L1, secreted by tumor cells, can suppress anti-tumor immunity and potentially contribute significantly to the failure of anti-PD-1 therapies (8). The targeted secretion of exosomes carrying immune checkpoint molecules into the synaptic cleft by cancer cells could increase the local concentration of these inhibitory molecules, thereby enhancing their capacity to inhibit CLs. Additionally, the synaptic secretion of EVs might associated with the surface exposure or release of molecules that antagonize CL cytotoxic activity. In this regard, melanoma cells have been shown to respond to CTL attack by a “secretory burst” of lysosomal/late endosomal vesicles, leading to cathepsin-mediated degradation of perforin and subsequent immune evasion (21). Another intriguing possibility is that tumor cell-derived synaptic EVs could carry death receptor ligands, such as FasL or TRAIL, which can induce apoptosis in immune cells (27). The localized delivery of such ligands through synaptic exosomes could enhance their potency by concentrating these death signals within the synaptic cleft, where they are most likely to engage with their corresponding receptors on immune effector cells. This would not only facilitate immune evasion by the tumor but could also undermine the efficacy of immunotherapeutic strategies that rely on the activation and persistence of CLs. However, these resistance mechanisms remain hypothetical and further research is required to isolate and characterize cancer cell-derived trans-synaptic EVs, which represents a technical challenge. While target cells and antigen-presenting cells can be effectively mimicked using functionalized supported lipid bilayers on glass substrates, enabling high-resolution imaging of the lymphocyte synaptic cortical plane and the isolation of lymphocyte-derived trans-synaptic EVs (7), there is currently no comparable model available for analyzing the cancer cell side of the IS. Characterizing the protein composition of EVs secreted by cancer cells during their interaction with NK cells (or CTLs) may be achieved through proteomics combined with labeling each cell population using different SILAC amino acids (28). Yet, the discrimination between EVs secreted in polarized and non-polarized manner remains a significant hurdle.

While cancer cells have been historically seen as passive during their interaction with CLs, accumulating evidence suggests that cancer cells can rapidly polarize and mobilize their defense arsenal towards the IS. Recently, polarized membrane trafficking has been identified as a potent synaptic defense mechanism employed by melanoma cells (20, 21). It has been demonstrated that ultrarapid calcium waves, triggered by the initial and non-lethal degranulation of a few lytic granules by CTLs, mobilize late endosomes/lysosomes to the tumor cell side of the IS and thereby facilitate local membrane repair and resistance to CTL-mediated cytotoxicity. Given that calcium signaling is a crucial regulator of actin cytoskeleton organization and dynamics (29), the role of perforin-dependent calcium entry in initiating synaptic remodeling of the actin cytoskeleton in cancer cells (16, 17) should be evaluated in future studies. In addition, whether the observed MVB mobilization and fusion at the cancer cell side of the IS promote reparative membrane turnover and resistance against NK cells per se warrants investigation. Supporting this hypothesis, a recent study has suggested that changes in the postsynaptic membrane composition, such as an accumulation of densely packed lipids, may protect breast cancer cells against NK cell-mediated lysis (30, 31). The origin of these membranous changes remains unknown, but locally elevated fusion of lysosomes and/or MVBs might contribute to this process.

During T cell activation, the polarization of MVBs to the IS has been shown to facilitate the local accumulation of clathrin, thereby promoting the recruitment of actin cytoskeleton regulators such as dynamin-2, the Arp2/3 complex, and CD2AP (32). Additionally, increasing evidence supports that clathrin not only regulates TCR internalization but also assists in the release of TCR-loaded vesicles (33). By analogy, synaptic polarization and fusion of MVBs at the tumor cell side of the IS may also serve dual purposes: facilitating the targeted secretion of immunomodulatory exosomes and orchestrating clathrin-dependent reorganization of the synaptic actin cytoskeleton. Notably, we have previously established the role of CDC42 and N-WASP, two critical regulators of the ARP2/3 complex, in mediating the actin response (16, 17). Therefore, potential links between MVB polarization, clathrin deposition, and Arp2/3 complex-mediated actin polymerization at the tumor cell side of the IS should be explored in future research.

The study presented in this brief report has certain limitations. The primary limitation is that the data were obtained from fixed samples, which introduces the possibility that the different synapses analyzed may not be at comparable stages. The high-throughput capabilities of imaging flow cytometry mitigate some of this concern. In addition, our previous live cell imaging analyses have shown that the AR is rapidly induced following conjugate formation, with no subsequent oscillations between AR+ and AR- phenotypes over time (16). Nevertheless, tracking actin and the CD63 vesicular compartment within individual cancer cells throughout the entire life cycle of the target cell-NK cell conjugate would provide further validation of our findings and, more crucially, determine which component polarizes first.

An essential future direction is to characterize the signaling pathways that initiate actin cytoskeleton remodeling at the tumor cell side of the IS. Elucidating these pathways will be crucial for a better understanding tumor cell resistance to NK cells and, potentially, CTLs. This knowledge will also pave the way for developing strategies to increase tumor cell susceptibility to immune cell cytotoxicity, thereby enhancing anti-tumor immunity and improving the efficacy of existing immunotherapies.

## Data Availability

The raw data supporting the conclusions of this article will be made available by the authors, without undue reservation.
