# Supplementary figures and images for: Is the tumor cell side of the immunological synapse a polarized secretory domain?
Source: Front Immunol. 2024 Sep 24;15:1452810. doi: 10.3389/fimmu.2024.1452810 (PMC11458426; doi:10.3389/fimmu.2024.1452810)

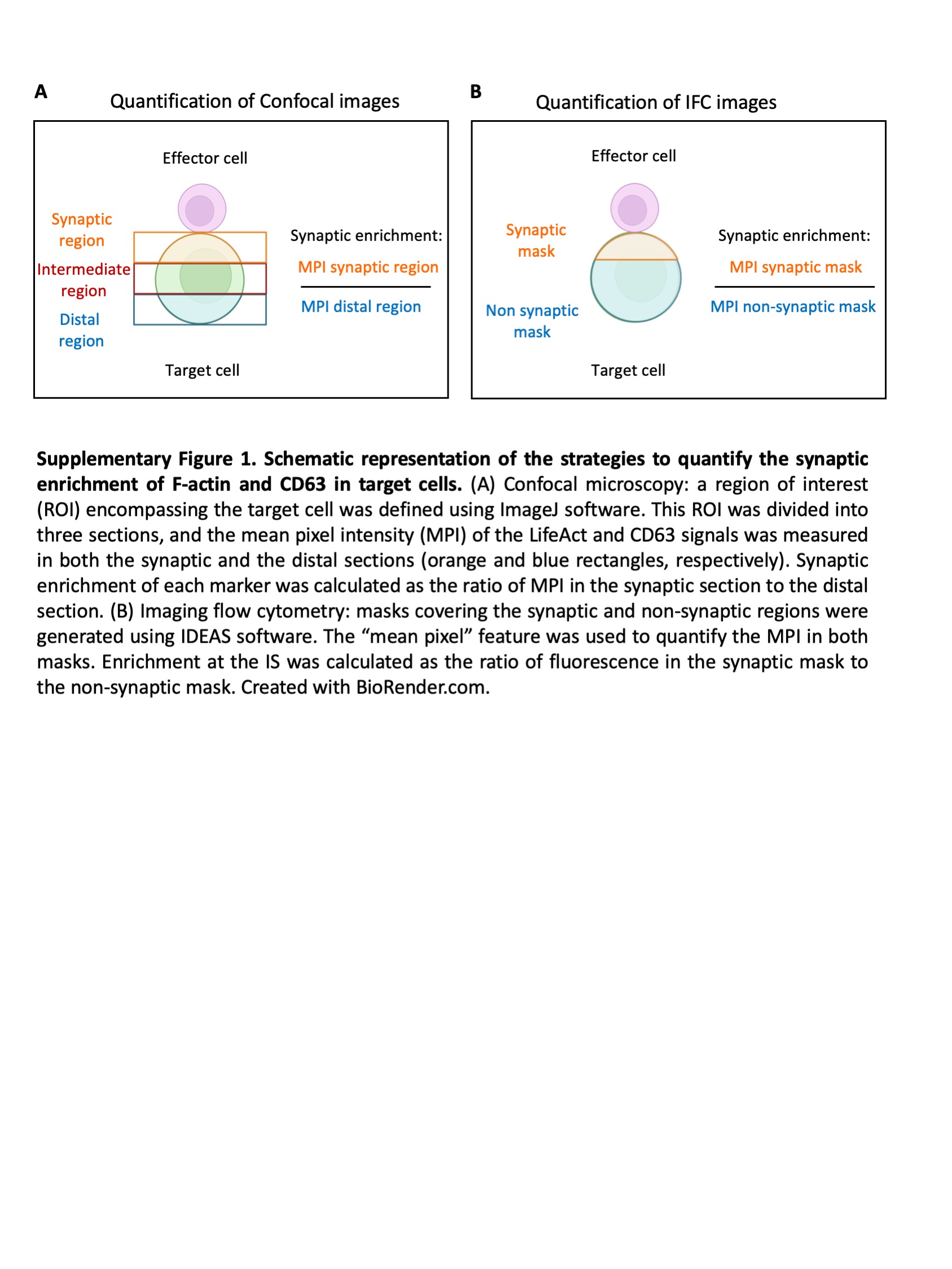

Supplement: Supplementary file 1 [file Image1.jpeg]

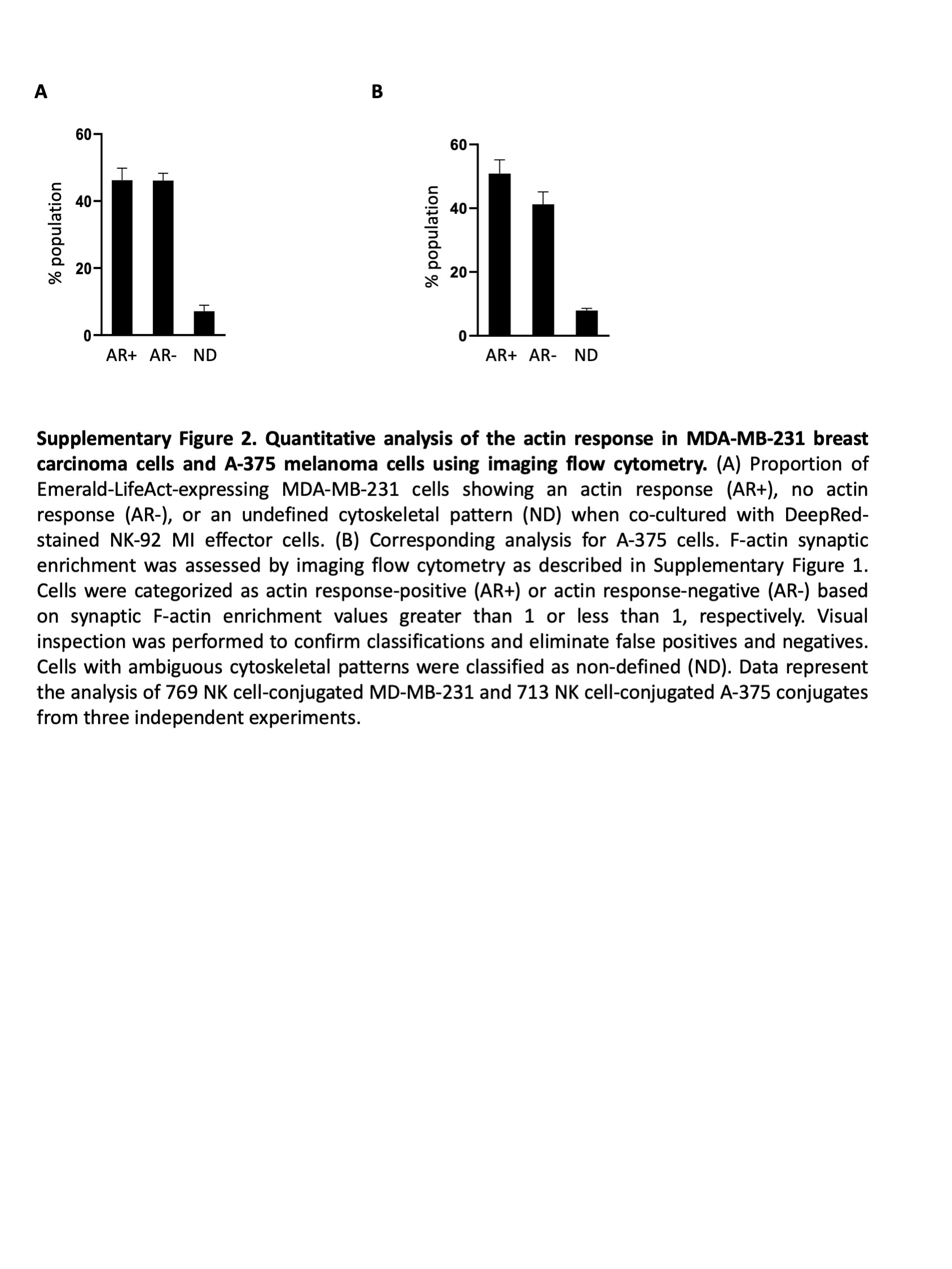

Supplement: Supplementary file 2 [file Image2.jpeg]

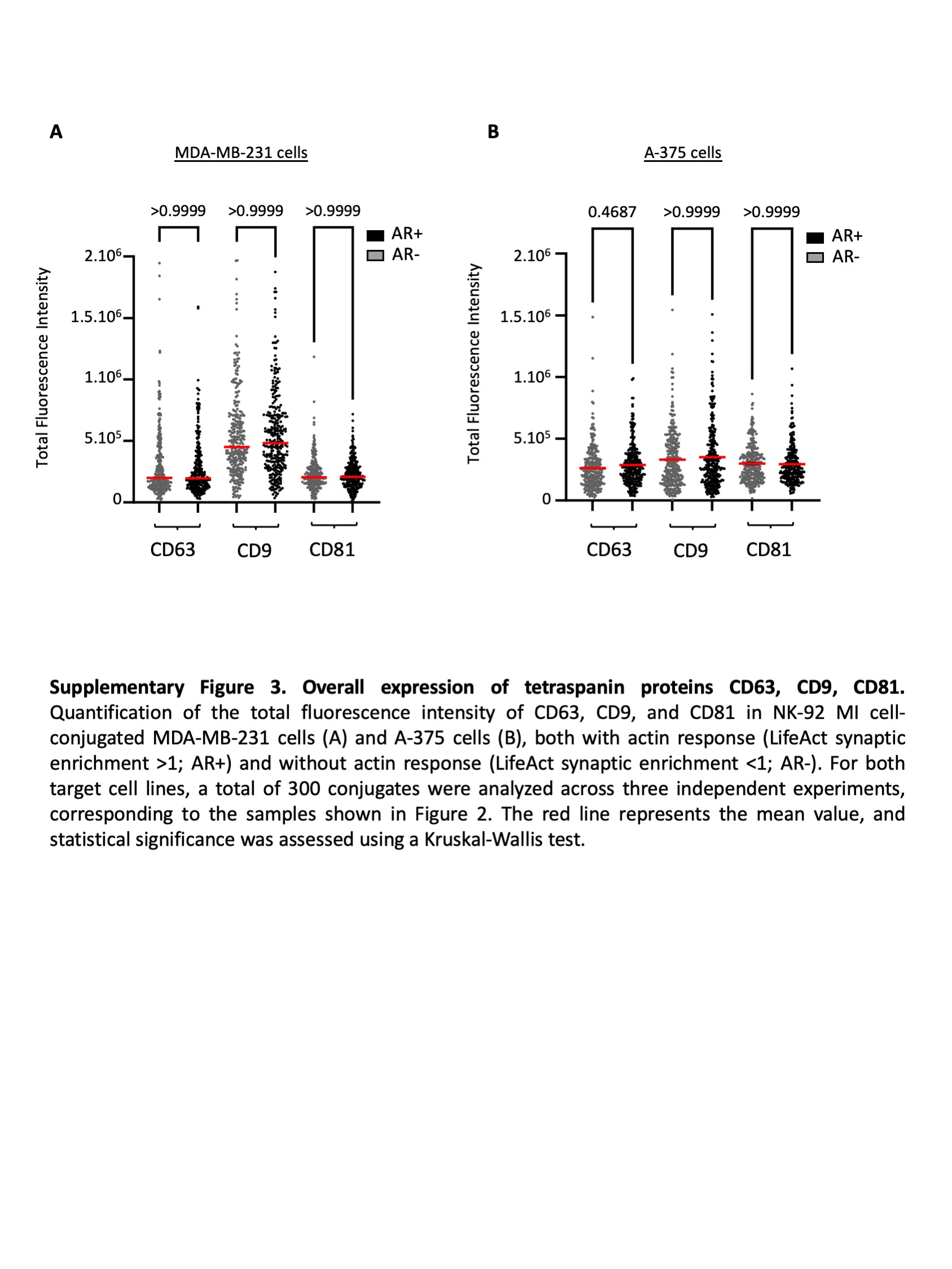

Supplement: Supplementary file 3 [file Image3.jpeg]

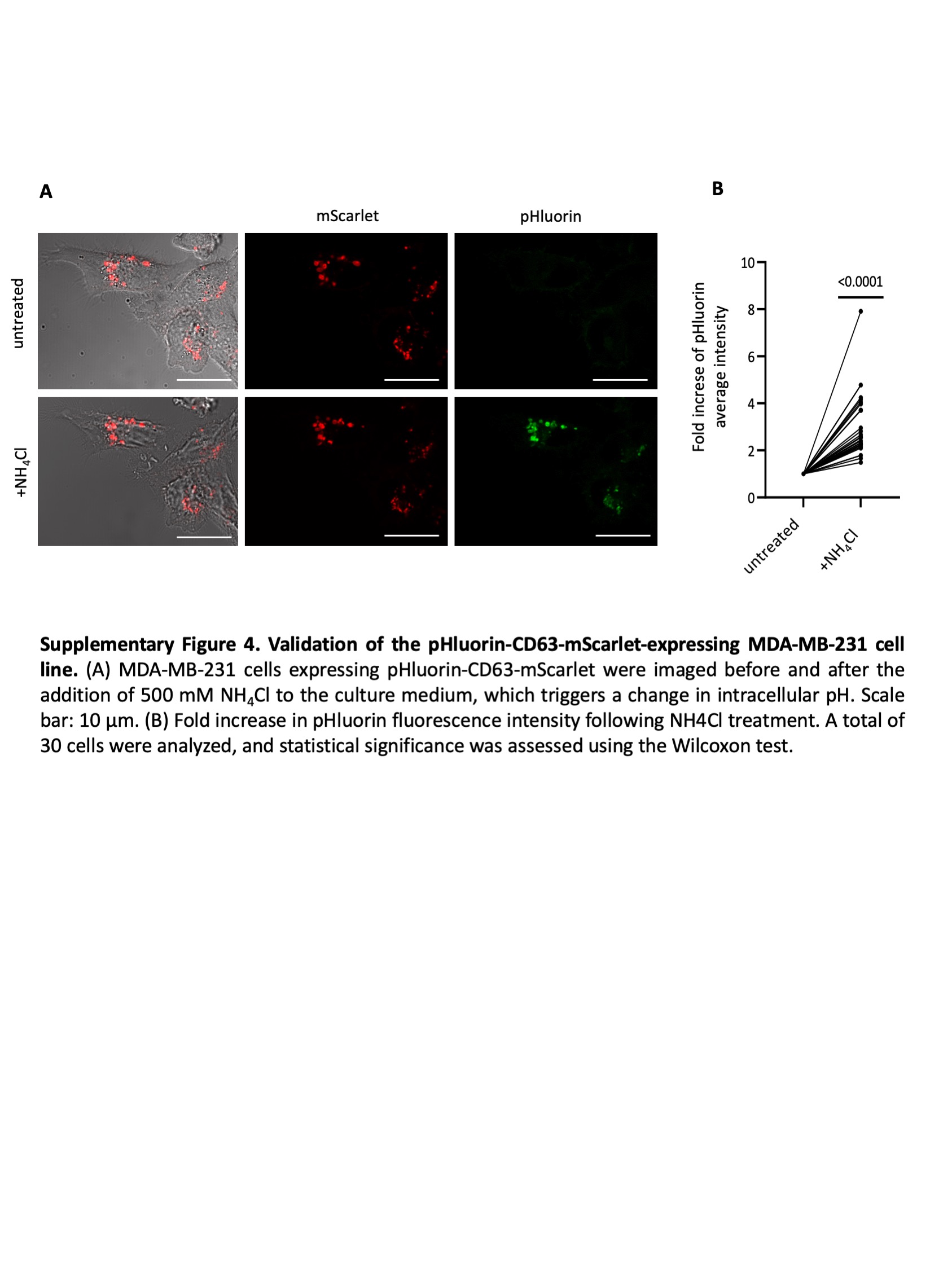

Supplement: Supplementary file 4 [file Image4.jpeg]
